# Biomineralization‐Inspired Membranization Toward Structural Enhancement of Coacervate Community

**DOI:** 10.1002/advs.202417832

**Published:** 2025-03-16

**Authors:** Chunyu Zhao, Xiaoliang Wang, Lianning Li, Hu Huang, Bingzhao Wu, Lei Zhang, Xin Huang

**Affiliations:** ^1^ School of Chemistry and Pharmaceutical Engineering Shandong First Medical University & Shandong Academy of Medical Sciences Taian Shandong 271016 China; ^2^ MIIT Key Laboratory of Critical Materials Technology for New Energy Conversion and Storage School of Chemistry and Chemical Engineering Harbin Institute of Technology Harbin Heilongjiang 150001 China; ^3^ School of Materials Science and Engineering Xinjiang University Urumchi Xinjiang 830000 China

**Keywords:** coacervates, liquid‐liquid phase separation, membranization, self‐assembly

## Abstract

The design and assembly of protocell models that can mimic the features and functions of life present a significant research challenge with the potential for far‐reaching impact. Inspired by the natural phenomenon of microbe‐induced mineralization, a way is developed to induce the spontaneous formation of mineralized membrane on the surface of coacervate droplets utilizing Fe^3+^ ions. In particular, the effect of Fe^3+^ ions on the microstructure of droplets at the molecular level is dissected by combining theoretical and experimental approaches. The reversible formation process of membrane can be regulated by redox reactions involving Fe^2+^/Fe^3+^ ions within the coacervate. The formation of mineralized membrane not only enhances the stability of the coacervate droplets and prevents aggregation and coalescence, but also allows the aggregation of adjacent droplets together. The membranized coacervate assemblages retain the inherent properties of biomolecule sequestration and enzyme catalysis, and also demonstrate excellent resistance to high temperatures and pressures as well as good stability for over 30 days. This study will offer a new platform for the assembly of coacervate‐based life‐like biomimetic systems, as well as enhance the understanding of the interactions underlying various biological phenomena at the molecular level.

## Graphical abstract

The spontaneous formation of a mineralized membrane on the surface of coacervate droplets is facilitated by Fe^3+^ ions and redox reaction, accompanied by the aggregation of the coacervate droplets. These membranized coacervate assemblages not only retain the capacity for biomolecule sequestration and enzyme catalysis but also exhibit enhanced structural stability.

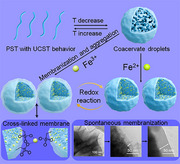

## 1 Introduction

Billions of years of evolution have shaped the diverse array of living systems present today. Living cells are composed of thousands of biomolecules and metabolites that interact within a complex network of cycles and reactions, enabling them to sense and adapt to their environment. However, many of the subtle complexities of these self‐assembled structures remain largely unknown. The bottom‐up approach focuses on the construction of micro/nanostructures using non‐living materials that replicate the characteristics of biomolecules, organelles, and cells. This process aims to utilize the interactions of simple non‐living chemical components to generate new functional components, with the eventual promise of creating cell‐like entities referred to as artificial cells (protocells, or synthetic cells).^[^ ^1^ ^]^ Examples of such protocells include lipid vesicles,^[^ ^2^ ^]^ polymersomes,^[^ ^3^ ^]^ colloidosomes,^[^ ^4^ ^]^ proteinosomes,^[^ ^5^ ^]^ coacervate droplets,^[^ ^6^ ^]^ and aqueous two‐phase systems droplets.^[^ ^7^ ^]^ These well‐designed protocell models have been widely used to study cell‐free gene transcription and translation,^[^ ^8^ ^]^ signaling communication and delivery,^[^ ^9^ ^]^ enzyme‐mediated cascades,^[^ ^10^ ^]^ growth, reproduction, and division.^[^ ^11^ ^]^ Consequently, the design and construction of artificial cells provide insights into fundamental questions regarding the understanding of life phenomena and the tracing of the origin of life, while also offering significant applications in fields such as biomedicine, bioengineering, and environmental science.

Coacervate droplets are one type of cell‐like compartment, which are formed spontaneously through liquid‐liquid phase separation (LLPS) of diverse molecules, driven by multivalent interactions including electrostatic attraction, cation‐π, π–π, hydrogen bonding, and hydrophobic interactions.^[^ ^6f^ ^]^ When these condensates are formed from two charge complementary polyelectrolytes, they are termed complex coacervation, or from a single component molecule in response to salt, pH, or temperature (*T*), denoted as simple or self‐coacervation.^[^ ^12^ ^]^ Notably, coacervate droplets, also known as biomolecular condensates in biology, have since been linked to a number of “membrane‐less organelles” or condensates in cells, including P‐bodies, nucleoli, and stress granules.^[^ ^13^ ^]^ These biomolecular condensates are capable of concentrating proteins and nucleic acids and have a variety of biological functions including RNA metabolism, ribosome biogenesis, and signal transduction. Because of their excellent functions, such as selective partitioning of biomolecules, enhancement of enzyme catalysis, and construction of cell‐like crowded environments, coacervate droplets have been broadly investigated as molecularly crowed protocell models both experimentally and theoretically. However, the membrane‐free coacervation system, which is open to the environment, exhibits liquid‐like properties such as rapid condensation and dissolution, fusion, and wetting, and is sensitive to salt/pH‐induced instability, which severely limits the in‐depth study of the microcompartment structure of the static and dynamic organization, as well as the more complex cytomimetic behaviors. To address this concern, on the one hand, components of various natures involved in the membrane construction are used for droplet interfacial self‐assembly, ranging from molecular amphiphiles^[^ ^14^ ^]^ to nano/micro‐scaled objects.^[^ ^6^, ^15^ ^]^ On the other hand, the introduction of external/internal stimuli (e.g., salt, *T*, pH, light, bioreaction) induces spontaneous morphological transitions within coacervate droplets that result in self‐membranization.^[^ ^16^ ^]^ The developed coacervate‐based protocells do utilize these different membrane components or membrane triggers to make the transition to membrane‐bounded microcompartments and achieve enhanced structural stability, while considering the complex components and stimuli in nature or living organisms, such as the widespread presence of inorganic metal ions, they are often neglected in the design of coacervate droplet structure and function.

Biomineralization is a widespread phenomenon in nature, a unique manifestation of the interaction between organic substrates and inorganic ions, and is closely associated with life activities.^[^ ^17^ ^]^ Inspired by this, we present a biomimetic stabilization strategy for coacervate droplets through Fe^3+^‐mediated interfacial membranization. By exploiting the Fe^3+^‐coordination capability of sulfonate‐functionalized zwitterionic polymers, we established a dynamic membranization‐aggregation coupling mechanism of coacervates regulated by the redox reactions involving Fe^2+^/Fe^3+^ ions. This approach not only resolves the inherent instability of coacervates but also endows dynamic environmental adaptability via redox‐triggered membrane reconfiguration. The membranized coacervate assemblages exhibited excellent resistance to high temperatures and pressures while preserving the inherent properties of biomolecule sequestration and enzyme catalysis, thereby bridging the gap between primitive coacervates and complex protocell systems. Our studies highlight the critical role of coacervate‐metal ion interactions, providing a conceptual framework for designing robust biomimetic materials with applications in synthetic cellular engineering and adaptive microreactor development.

## 2 Results and Discussion

### 2.1 Simple Self‐Coacervation of Zwitterionic Polymer

**Figure 1:**
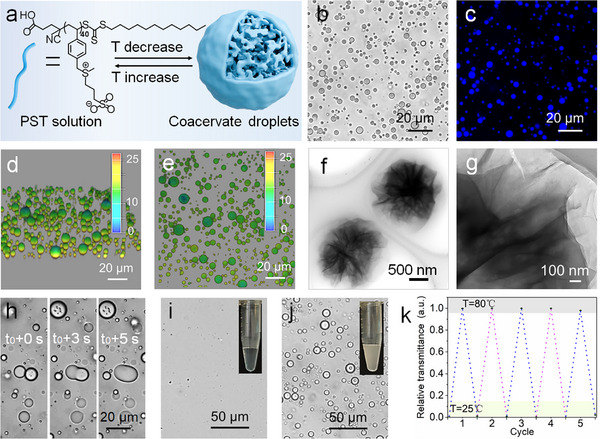
The morphology and physical properties of the coacervate droplets. a) Schematic diagram of temperature‐triggered self‐coacervation of PST. b) Optical and c) fluorescence microscopy images of coacervate droplets. d) 3D reconstructed CLSM image and e) the corresponding overhead view image of coacervate droplets. f) The HR‐TEM images of the coacervate droplets and g) the enlarged view. h) Time‐lapse microscopy images of coacervate dispersion. i, j) The morphology of the coacervate droplets at (i) 80 °C and j) 20 °C, respectively. k) Reversible phase separation behavior of coacervate droplets. Error bars represent the standard deviations of three measurements.

Well‐known examples of biologically relevant thermoresponsive polypeptides or protein‐based polymers that form liquid‐like coacervates by regulating temperature are elastin‐like polypeptides and resilin‐like peptides. Inspired by the architecture of thermoresponsive polypeptides or proteins, we identified and synthesized a reference zwitterionic polymer (PST) by reversible addition‐fragmentation chain‐transfer (RAFT) polymerization (Mn 11 840 g mol^−1^, PDI 1.14, monomer repeat units 40, Figure S1–S4, Supporting Information). Interestingly, PST exhibited a thermally induced phase transition in aqueous media, remaining insoluble at ambient temperatures but dissolving completely upon heating, a characteristic feature of UCST behavior. The phase transition temperature increased with polymer concentration (1–20 mg mL^−1^), driven by enhanced interchain interactions, while urea disrupted hydrogen bonding and lowered the transition threshold, confirming solvation and hydrogen bonding as primary UCST drivers (Figure S5, Supporting Information). Observation of the PST turbid solution (20 mg mL^−1^ in PBS, 10 mm, pH 5.0) by optical microscopy and confocal fluorescence microscopy (CLSM) at 20 °C revealed that the polymer spontaneously coalesced into discrete spherical droplets with a diameter of ≈2.88 µm (**Figure** **1**a–e, Figure S6, Supporting Information). The blue fluorescence observed in the CLSM images (Figure 1c) originated from the intrinsic fluorescence of the polymer, a characteristic attributed to the π‐conjugated phenyl groups within its side chains (Figure S7, Supporting Information). Furthermore, the high‐resolution transmission electron microscopy (HR‐TEM) images of the coacervate droplets revealed a low density and loose, sponge‐like microstructure (Figure 1f,g). As illustrated in Figure 1h, these formed droplets readily coalesced, deformed, and wetted glass slides, demonstrating liquid‐like properties. Besides, the temperature‐controlled dynamics were evidenced by the repeated induction of phase transitions between the solution and coacervate droplet states through temperature adjustments (Figure 1i‐k). We attributed this reversible self‐coacervation phenomenon to the charge‐charge interactions and hydrogen bonding. Subsequently, the coacervate droplets exhibited stability under salt concentrations (up to 250 mm NaCl, Figure 5g), high urea concentrations (up to 4 m urea, Figure S8, Supporting Information), and a wide pH range (2–11) (Figure S9, Supporting Information). Furthermore, the coacervate droplets exhibited no significant sensitivity to the addition of 1,6‐hexanediol, which demonstrated that hydrophobic interactions contributed minimally to the self‐coacervation process (Figure S10, Supporting Information). Notably, the formation of these coacervate droplets necessitated a concentration threshold. The minimum concentration of PST required for self‐coacervation at 20 °C was 2.5 mg mL^−1^ in PBS solution (10 mm, pH 5.0), and the droplet diameter was statistically concentration‐dependent (ranging from 2.5 to 30 mg mL^−1^), as evident from turbidity measurement and microscopic studies (Figure S6, Supporting Information). The PST turbid solution (20 mg mL^−1^ in PBS, 10 mm, pH 5.0) was chosen for subsequent studies due to its favorable size distribution and droplet size.

### 2.2 Membranization of Coacervate Droplets Mediated by Fe^3+^ ions

**Figure 2:**
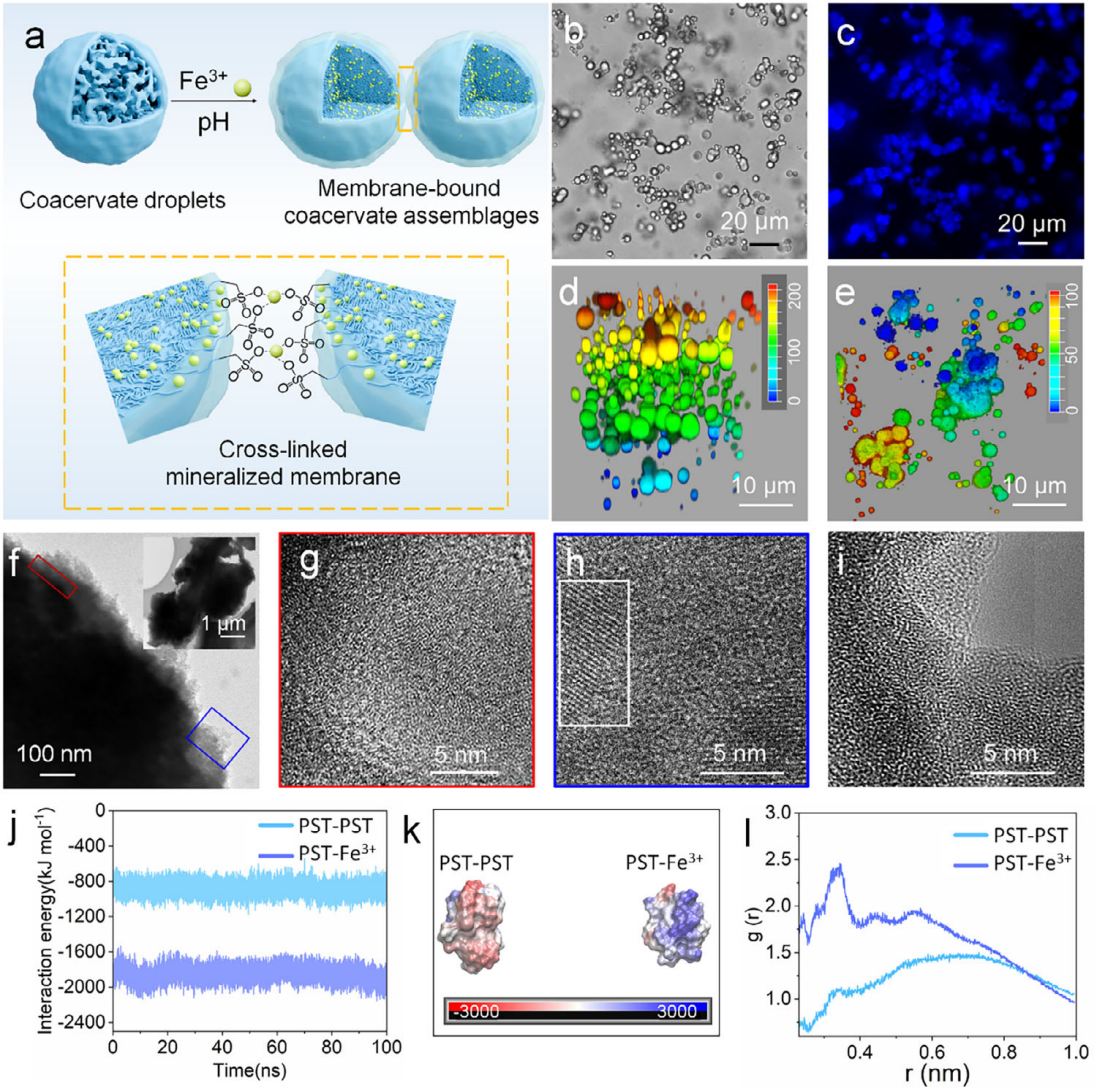
Fe^3+^ ions‐mediated membranization of coacervate droplets. a) Schematic diagram of the membranization and aggregation of coacervate droplets. b) Optical and c) fluorescence microscopy images of the membranized coacervate assemblages (intrinsic fluorescence). d) 3D reconstructed CLSM image and e) the corresponding overhead view image of the coacervate assemblages. f–h) The HR‐TEM images of the membranized coacervate assemblages, (g) enlarged view corresponding to the red frame in (f), (h) enlarged view corresponding to the blue frame in (f). i) The HR‐TEM images of the coacervate droplets. j) The interaction energies between PST and Fe^3+^ ions, and PST with PST chain. k) The final surface electrostatic potential of the PST and PST mixed cluster (left), the PST and Fe^3+^ ions mixed cluster (right). The color scale represents the range of the surface electrostatic potential, where the minimum (red) and maximum (blue) values was −3 and 3 kT e^−1^, respectively. l) Radial distribution functions, g(r), of Fe^3+^ ions and PST around the center of the clusters. The results are derived from 100 ns simulations.

In nature, living organisms can form organic‐inorganic composites through biomineralization, which facilitates functional evolution and enhances adaptability to the environment. Inspired by this process, we aimed to improve the stability of droplets by inducing the formation of a mineralized membrane on the surface of coacervate droplets using metal ions. The PST solutions (20 mg mL^−1^, PBS 10 mm, pH 5.0) were mixed with Fe^3+^ ions (100 mm, H_2_O) in a molar ratio of 1:1.8 at 20 °C. Optical and fluorescence microscopy images, as well as the 3D reconstructed CLSM images, demonstrated that the spatially hierarchical coacervate assemblages were formed by multiple tightly connected coacervate droplets (**Figure** **2**a‐e). In situ observations revealed that there was no fusion between adjacent droplets within 1 h (Figure S11, Supporting Information), suggesting improved structural stability of coacervate droplets. Besides, the addition of Fe^3^⁺ ions significantly elevated the zeta potential of the coacervate droplets from +3.6 to +16.6 mV, indicating enhanced electrostatic stabilization (Figure S12, Supporting Information). To further investigate the reasons behind the changes in stability and morphology of the coacervate droplets, we examined the microstructure of the coacervate using HR‐TEM. In contrast to the disordered amorphous structure of the coacervate droplets (Figure 2i), the detailed characterization confirmed that the inner layer structure of the coacervate assemblages had undergone significant changes, with molecular chains becoming more tightly wound. Furthermore, the edges of the interconnected droplet structure exhibited regionally distinct lattice fringes, indicating a transition from an amorphous to a crystalline structure (Figure 2f–h). The corresponding energy‐dispersive X‐ray spectroscopy (EDX) elemental mapping further confirmed the presence of Fe in the coacervate assemblages compared to the coacervate droplets (Figure S13, Supporting Information). Moreover, the partial enlargement of the blue area (Figure 2f, blue frame) and the corresponding EDX elemental mapping (Figure S14, Supporting Information) demonstrated the co‐presence of polymers and Fe, akin to the co‐precipitation of organic matter with Fe ions during the biomineralization process. We defined the region of amorphous‐to‐crystalline transition as the mineralized membrane. Given these findings, we conjectured that the variations in the internal molecular chain aggregation state structure and droplets morphology were closely related to the incorporated Fe^3+^ ions, specifically the complexation of Fe^3+^ ions with sulfonic acid groups, accompanied by the deposition of Fe^3+^ ions. This may enhance the structural stability of the coacervate droplets. To investigate this further, we compared the infrared spectra of free PST with the PST‐Fe^3+^ complex. The appearance of a new band at 594.2 cm^−1^, attributable to the vibration of the Fe─O bond, confirmed the complex formation, while the frequencies of ligand functions not involved in complexation remained unchanged (Figure S15, Supporting Information).^[^ ^18^ ^]^ Besides, the X‐ray photoelectron spectroscopy (XPS) spectrum revealed the binding energy of Fe^3+^ ions within the coacervate assemblages (Figure S16, Supporting Information). Thus, the internal molecular chain aggregation state structure and droplet morphology were closely related to the incorporated Fe^3+^ ions.

To further confirm the aforementioned conclusions, we performed molecular dynamics (MD) simulations to explore the interactions between PST and Fe^3+^ ions (Figure 2j–l). As shown in Figure 2j, the average interaction energy between PST and Fe^3+^ ions (−1850 kJ mol^−1^) was significantly stronger than that of PST with PST (−883 kJ mol^−1^). This indicated that PST had a much higher propensity to bind with Fe^3+^ ions, resulting in a stronger interaction force and a more stable structure. Additionally, we also performed all‐atom molecular dynamics simulations to construct the mixed clusters of PST with PST (PST‐PST), and PST with Fe^3+^ ions (PST‐Fe^3+^), respectively (Figure S17, Supporting Information). Figure 2k revealed that the surface of the PST‐PST mixed cluster was mainly negatively charged, whereas the surface of the PST‐Fe^3+^ mixed cluster exhibited a distribution of positively charged Fe^3+^ ions. The radial distribution functions (RDFs) of Fe^3+^ ions and PST around the center of the two types of clusters indicated that in compared to PST‐PST, the presence of a distinct sharp diffraction peak at r = 0.34 nm for PST‐Fe^3+^ suggested an ordered structure within a specific range, as well as a closer binding of Fe^3+^ ions to PST (Figure 2l). Importantly, all these simulations agreed with our experimental results.

**Figure 3:**
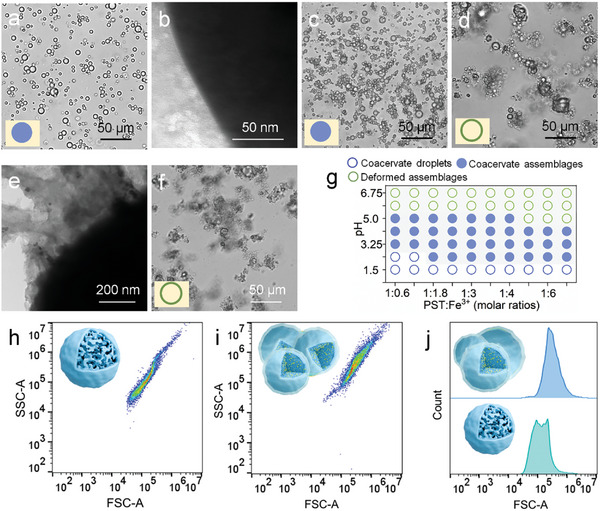
Membranization of coacervate droplets under the different concentrations of Fe^3+^ ions and pH. a) The morphology of the coacervate droplets with Fe^3+^ ions (PST: Fe^3+^, molar ratio, 1:0.6) and b) the corresponding HR‐TEM images. c–e) The morphology of the coacervate droplets with different concentrations of Fe^3+^ ions (i.e., PST: Fe^3+^, molar ratios), (c) 1:2.4 and (d) 1:4 and (e) the corresponding HR‐TEM images of (d). f) The morphology of the coacervate droplets with Fe^3+^ ions at pH 6.0 (PST: Fe^3+^, molar ratio, 1:2.4). g) Phase diagrams of the coacervate assemblages as a function of pH and Fe^3+^ ions concentrations. h–j) Fluorescence‐activated cell‐sorting study of the formation process of the coacervate assemblages. (h) 2D pseudocolor plots for coacervate droplets, (i) 2D pseudocolor plots for the coacervate assemblages (PST: Fe^3+^, molar ratio, 1:2.4, pH 5.0), and (j) corresponding plots of counts against FSC‐A values for droplets and coacervate assemblages.

Given that the concentration of Fe^3+^ ions and pH are the key factors influencing the complexation and deposition processes between Fe^3+^ ions and PST, which might impact the formation of mineralized membrane, we further investigated the effects on the structural stability and morphology of the coacervate droplets. Notably, optical microscopy images showed that as the concentration of Fe^3+^ ions increased in the system, the aggregation of droplets also increased at pH 5.0 (**Figure** **3**a–c). Specifically, when the coacervate droplets contained a low concentration of Fe^3+^ ions (PST: Fe^3+^, molar ratio, 1:0.6), only a small number of coacervate assemblages were observed in solution (Figure 3a), and the corresponding HR‐TEM image indicated the presence of an extremely thin mineralized membrane covering the surface of droplets (Figure 3b). In contrast, as the concentration of Fe^3+^ ions increased (PST: Fe^3+^, molar ratio, below 1:4), the structure of coacervate assemblages became deformed due to the deposition of excess Fe^3+^ ions (Figure 3d), with the HR‐TEM image revealing the droplet edges adorned with large mineral crystals (Figure 3e). Furthermore, our results indicated that no coacervate assemblages were observed at lower solution pH values (below 2.0) across a broad range of Fe^3+^ ions concentration (PST: Fe^3+^, molar ratios, 1:3 or 1:6) (Figure S18, Supporting Information). This phenomenon primarily occurred because, under these conditions, Fe^3+^ ions were unable to deposit and could only form 1:1 complex with PST. The observation was further corroborated by the complexation rate of Fe^3+^ ions with PST (PST: Fe^3+^, molar ratios, 1:0.6–1:0.3), which exceeded 98% at pH 2.0 (Figure S19, Supporting Information). The coacervate assemblages were observed over a wide range of Fe^3+^ ion concentrations while altering the pH from 2.3 to 5.0, resulting from complexation and the deposition of Fe^3+^ ions (Figure 3g). However, when the pH exceeded 5.5, the morphology of the coacervate assemblages was deformed, primarily due to the deposition of Fe^3+^ ions (Figure 3f). Taken together, these findings suggested that the formation of mineralized membranes and coacervate assemblages could be achieved by modulating the concentration of Fe^3+^ ions and pH within a certain threshold. To be of interest, the coacervate assemblages could not be dispersed by the addition of PBS buffer (Movie S1, Supporting Information) and gentle stirring (Movie S2, Supporting Information), as the mineralized membranes of the droplets were cross‐linked, maintaining the integrity of the droplets without fusion. Therefore, the appearance of the coacervate assemblages also implied the formation of mineralized membranes. In addition, fluorescence‐activated cell sorting (FACS) analysis of the coacervate droplets and the assemblages displayed distinguishable 2D pseudo‐color dot plots, attributed to the more complex granularity and larger particle size of the coacervate assemblages (Figure 3h–j).

### 2.3 Membranization and Reversible Aggregation of Coacervate Droplets by Redox Reactions

**Figure 4:**
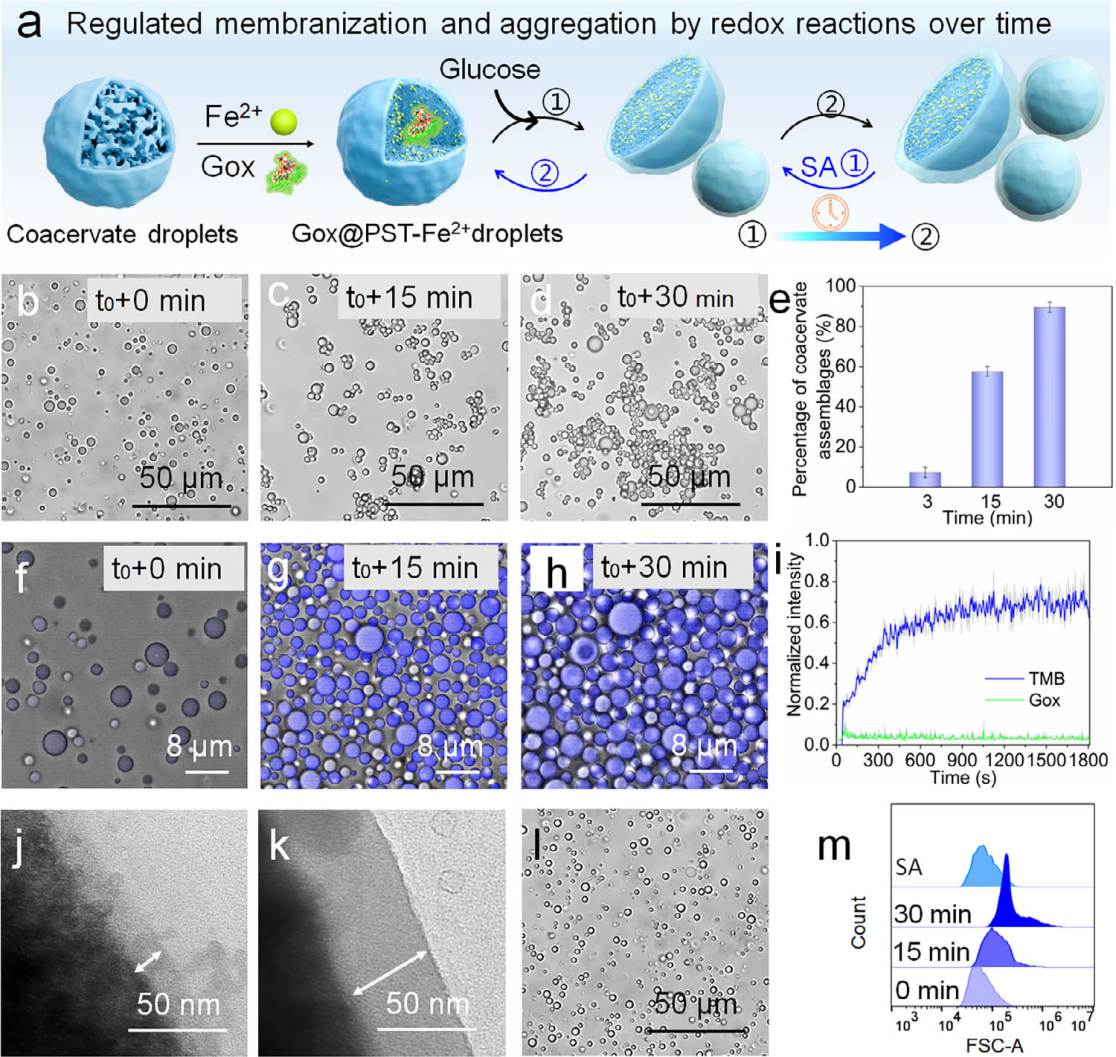
Reversible membranization of coacervate droplets by redox reactions. a) Schematic illustration for the membranization and aggregation of the coacervate droplets by establishing redox reactions involving enzymes and Fe^3+^/Fe^2+^ ions. b–d) Time‐dependent optical microscopy morphology images of the transformation from coacervate droplets to coacervate assemblages, (b) t_0_+0 min, (c) t_0_+15 min, (d) t_0_+30 min. e) Time‐dependent percentage of coacervate assemblages in solution. ≈300 droplets were measured with Image J. Error bars represent standard deviations of three measurements. f–h) Time‐dependent CLSM morphology images of the transformation from coacervate droplets to coacervate assemblages, (f) t_0_+0 min, (g) t_0_+15 min, (h) t_0_+30 min, and i) corresponding fluorescence intensity of TMB (blue line) and RITC‐labelled Gox (green line) changes with time. Error bars represent the standard deviations of three measurements. j, k) Time‐dependent HR‐TEM images of the coacervate droplets with Fe^2+^ ions, (j) t_0_+15 min, (k) t_0_+30 min. l) The optical microscopy morphology image of the re‐dispersed coacervate droplets by adding the SA into the PST‐Fe^2+^ coacervate droplets (t_0_+15 min). m) The corresponding plots of counts against FSC‐A values for PST‐Fe^2+^ coacervate droplets at different times and for re‐dispersed droplets.

Redox reactions play an important role in biomineralization. Microorganisms can derive energy from the redox cycling of variable‐valent elements within the mineral structure, thereby promoting mineral formation. Building on this premise, we aimed to programmatically regulate the membranization and aggregation of the coacervate droplets by establishing redox reactions involving enzymes, Fe^2+^ ions, and Fe^3+^ ions (**Figure** **4**a). It was found that the coacervate droplet solutions (pH 5.0) containing different concentrations of Fe^2+^ ions did not result in the formation of coacervate assemblages, and the droplets coalesced and wetted glass slides (Figure S20, Supporting Information). Subsequently, the RITC‐labeled GOx was encapsulated into coacervate droplets, and glucose was added to the solution to trigger the production of hydrogen peroxide (H_2_O_2_). The resulting H_2_O_2_ oxidized Fe^2+^ ions to Fe^3+^ ions, while the accompanying hydroxyl radicals (·OH) were monitored using 3,3′,5,5′‐tetramethylbenzidine (TMB). As expected, the time‐dependent optical microscopy and CLSM images confirmed an increase in the number of aggregated droplets (Figure 4b–d and Figure 4f–h, Movie S3, Supporting Information). The corresponding time‐dependent microscopy image statistics indicated that over 85% of the droplets had aggregated into assemblages after 30 min (Figure 4e). Moreover, as shown in Figure 4i, the fluorescence intensity of TMB, which increased over time, provided evidence for the occurrence of redox reactions within the system, as well as the stable presence of the encapsulated RITC‐labeled GOx in the droplet (Figure S21, Supporting Information). Further analysis of the microstructure of coacervate droplets with Fe^2+^ ions, as revealed by the HR‐TEM, indicated a denser interior of the coacervate assemblages compared to those without Fe^2+^ ions (Figure 1g). It was worth noting that the thickness of mineralized membrane on the surface of coacervate droplets also increased progressively over time, with measurements indicating that the membrane thickness at 30 min was about three times that at 15 min (Figure 4j,k). Moreover, the membrane formed by redox reaction was more regular compared to the inhomogeneous membrane (Figure 2f) formed by the direct addition of Fe^3+^ ions to the droplets, which may be related to the release kinetics, spatial distribution and interfacial environment of the Fe^3+^ ions.^[^ ^19^ ^]^ Corresponding EDX elemental mapping demonstrated the presence and content of elemental C, O, S, and Fe in the coacervate droplets with Fe^2+^ ions (Figure S22, Supporting Information). The occurrence and oxidation state of Fe in the coacervate assemblages after 15 min were determined by XPS (Figure S23, Supporting Information). Characteristic peaks at 710 and 711.89 eV were identified, corresponding to Fe 2p3/2 of Fe^2+^ ions and Fe^3+^ ions, respectively, indicating that some Fe^2+^ ions had been oxidized to Fe^3+^ ions. Furthermore, time‐dependent plots of forward‐light‐scattered area (FSC‐A) versus side‐light‐scattered area (SSC‐A), derived from fluorescence‐activated cell sorting measurements over 30 min, demonstrated a progressive emergence of a peak in the maximum FSC‐A count (Figure 4m, Figure S24a–c, Supporting Information), attributed to an increasing number of coacervate assemblages. Interestingly, the coacervate assemblages could be re‐dispersed into individual droplets by reducing Fe^3+^ ions to Fe^2+^ ions through the addition of sodium ascorbate (SA) (Figure 4l, Movie S4, Supporting Information). Moreover, the FSC‐A count of the re‐dispersed coacervate assemblages was comparable to that of the initial coacervate droplets (Figure 4m, Figure S24d, Supporting Information). The aggregation and dispersion of coacervate droplets primarily resulted from the varying pH environments required for the deposition of Fe^3+^ ions and Fe^2+^ ions, because Fe^2+^ ions facilitated the formation of coacervate assemblages at a pH of 8.0 (Figure S25, Supporting Information). Consequently, the process of formation and thickness of the mineralized membrane over time could be regulated by establishing redox reactions involving Fe^3+^/Fe^2+^ ions in the system.

### 2.4 Biophysical Properties of the Membranized Coacervate Assemblages

**Figure 5:**
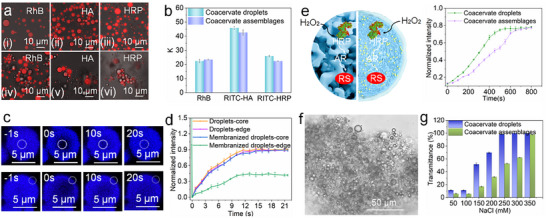
Properties of membranized coacervate assemblages. a) The CLSM images of the sequestration of RhB, RITC‐HA (MW = 50 kDa), and RITC‐HRP in (i, ii, iii) coacervate droplets and (iv, v, vi) coacervate assemblages, respectively, and b) the corresponding diagram of sequestration coefficient (K). Data were normalized to correct for variations in background fluorescence intensity. Error bars represent the standard deviations of three measurements. c) Time series of CLSM images after photobleaching the area (the core region or the edge region) in coacervate assemblages (intrinsic fluorescence), and (d) corresponding fluorescence recovery curves in bleached areas. Error bars represent the standard deviations of three measurements. e) Schematic diagram of the enzyme catalytic reactions within coacervate droplets or coacervate assemblages. Conversion of AR into red fluorescent RS by H_2_O_2_‐mediated oxidation catalyzed by HRP. The line chart showed the change in red fluorescence intensity over time. f) Optical microscopy morphology of the coacervate assemblages after centrifugation (6000 rpm, 10 min). g) Turbidity of coacervate droplets and coacervate assemblages at different salt concentrations, respectively.

A key feature of protocells that mimic living cells is their ability to recruit a variety of biomolecules. To this end, we further explored the effect of mineralized membranes on the properties of droplets. As shown in **Figure** **5**a,b, the constructed membranized coacervate assemblages could selectively sequester molecules, including rhodamin B (RhB), sodium hyaluronate (HA), and horseradish peroxidase (HRP), without a significant change in sequestration capacity compared to that of coacervate droplets. Fluorescence recovery after photobleaching (FRAP) measurements indicated the internal fluidity of membranized coacervate assemblages remained comparable to that of membrane‐free coacervate droplets, with fluorescence recovering 90% of its original value within 20 s. This indicated that the membranized coacervate interior retained liquid‐like properties. In contrast, at the mineralized membrane interface, only 40% fluorescence recovery was observed after 21 s, suggesting that the crystalline structure of the mineralized membrane restricted molecular mobility (Figure 5c–d, Figure S26, Supporting Information). This observation reflected the ability of membranized coacervates to maintain fundamental biological functions. Many life activities depend on the catalytic reaction of enzymes, which are essential for the normal metabolism and growth of organisms. To investigate this further, horseradish peroxidase (HRP, 0.2 µg mL^−1^) was encapsulated within either coacervate or membranized coacervate assemblages. In the presence of H_2_O_2_, Amplex Red (AR) was converted into resorufin (RS) with red fluorescence, which could be detected by CLSM (Figure S27, Supporting Information). The catalytic ability of membranized coacervate assemblages was comparable to that of coacervate droplets; however, the membranized systems exhibited a slightly lower catalytic rate, which we attributed to the inhibitory effect of FeCl_3_ on the reaction (Figure 5e, Figure S28, Supporting Information). Statistical analysis of turbidity across varying temperatures revealed that membranized coacervate assemblages demonstrated significantly enhanced thermal stability compared to membrane‐free coacervate droplets (Figure S29, Supporting Information). As illustrated in Figure 5f, the membranized coacervate assemblages were denser after high‐speed centrifugation (6000 rpm, 10 min), yet no coalescence occurred between droplets. So, we believed that the formed mineralized membranes were sufficiently flexible to withstand localized deformation, in contrast to coacervate droplets, which coalesced under lower centrifugation conditions (3000 rpm, 5 min) (Figure S30, Supporting Information). Statistical analysis revealed that the membranized coacervate assemblages could remain 50% of their initial turbidity in sodium chloride solution (250 mM NaCl) and the rate of diffusion of molecules in the droplets was rapid (Figure S31, Supporting Information), while the coacervate droplets completely dissociated into the solution (Figure 5g). Currently, we found that the dense, cross‐linked mineralized membranes could form a barrier that prevented direct contact and coalescence between adjacent coacervate droplets, allowing the coacervate assemblages to be maintained for at least 30 days (Figure S32, Supporting Information).

## 3 Conclusion

In this study, we developed a method for the spontaneous formation of a mineralized membrane on the surface of coacervate droplets, mediated by Fe^3+^ ions, to enhance the structural stability of the droplets. Additionally, we demonstrated the aggregation phenomenon that occurred between droplets during the membranization process. The well‐defined UCST‐type thermoresponsiveness PST, characterized by sulfonate groups, can spontaneously condense into coacervate droplets as the temperature decreased. The formation of the mineralized membrane on the surface of the coacervate droplets depended on the concentration of Fe^3+^ ions and pH, with the cross‐linked mineralized membrane facilitating the aggregation of adjacent droplets. Especially given that the microbial‐induced mineralization in nature is closely related to the redox reactions of organisms, we further showed the reversible formation process and thickness of the membrane could be controlled by redox reactions within the system. The membranized coacervate assemblages retained good biomolecule sequestration and enzyme catalysis and demonstrated excellent performance in extreme environments including high temperatures, high pressures, and long‐term storage (over 30 days). This provides insights into the adaptability of protocells on early Earth. We expect that our findings will offer a new model for the assembly of coacervate‐based life‐like biomimetic systems. Moreover, as a proof‐of‐concept, we envision that our results will contribute positively to the understanding of the phenomena and mechanisms of biomineralization in nature, enhance the correlation between organic matrices and inorganic materials, and aid in the design of biomimetic smart materials.

## 4 Experimental Section

### Materials

Sodium thiomethoxide (95%, Sigma–Aldrich), tetrahydrofuran (THF, 99.5%, extra dry, with molecular sieves, stabilized with BHT, Energy Chemical), 4‐vinylbenzyl chloride, 1,3‐propanesultone, anhydrous acetonitrile, butylated hydroxytoluene (BHT, 99%, Aladdin), ether (Aladdin), mercaptothiazoline‐activated trithiol‐RAFT agent (95%, Sigma–Aldrich), 2,2′‐Azobis(2‐methylpropionitrile) (AIBN, Energy Chemical) was recrystallized from methanol, sodium nitrate(NaNO_3_, Energy Chemical), hyaluronic acid sodium salt (HA, M_W_ = 50 000, Bomeibio), Rhodamine B isothiocyanate (RITC, Sigma–Aldrich), glucose oxidase from spergillus niger (GOx, Sigma–Aldrich), peroxidase from horseradish (HRP, Sigma–Aldrich), glucose (≥99.5%, Sigma–Aldrich), ferrous (II) chloride tetrahydrate (FeCl_2_·4H_2_O, ≥99%, Sigma–Aldrich), Iron (III) chloride hexahydrate (FeCl_3_·6H_2_O, ≥99%, Sigma–Aldrich), 1,10‐phenanthroline hydrate (≥99%, Aladdin), hydroxylamine hydrochloride (≥99%, Aladdin), sodium ascorbate (SA, ≥99%, Aladdin), acetic acid (≥99.5%, Aladdin), sodium acetate(≥99%, Aladdin), 3,3′,5,5′‐tetramethylbenzidine (TMB, ≥98%, Sigma–Aldrich). Deionized water (specific resistance of 18.2 MΩ∙cm, Milli‐Q Reference, Millipore, USA) was used in all the experiments.

### Characterization Methods

Optical and fluorescence microscopy was performed on a Leica DMI8 manual inverted fluorescence microscope at 63x magnification. Confocal images were obtained on a Leica TCS SP8 confocal laser scanning microscope attached to a Leica DMI 6000 inverted epifluorescence microscope. Fluorescence activated cell sorter (FACS) analysis was undertaken on a Flow cytometry (Beckman Coulter, Inc.). High‐resolution transmission electron microscopy (HR‐TEM) analysis was undertaken on a FEI Tecnai G2 F20. Samples were prepared by adding one drop of solution onto a 200‐mesh carbon film‐coated copper grid followed by drying in a vacuum for one day. Fourier Transform infrared spectroscopy (FTIR) measurements were performed on PerkinElmer spectrometer with a LiTaO3 detector (Spectrum Two, USA). X‐ray photoelectron spectroscopy (XPS) spectrum analysis was undertaken on a X‐ray photoelectron Spectrometer (Thermo Scientific K‐Alpha). Dynamic light scattering (DLS) and zeta potential measurements were performed using a Malvern Zetasizer Nano‐ZS with either low volume glass cuvettes (DLS) or folded capillary cells (zeta potential). UV–vis spectra were measured on a PerkinElmer spectrophotometer (Lambda 750S, USA). ^1^H NMR spectra were recorded using a BRUKER ADVANCED spectrometer operating at 400 MHz at room temperature. Fluorescence spectra were recorded on the Hitachi F‐4600 FL Spectrophotometer.

### Synthesis of 4‐vinylbenzyl Methyl Sulfide

4‐vinylbenzyl methyl sulfide was synthesized according to the previously reported method.^[^ ^20^ ^]^ Sodium thiomethoxide (3.4 g, 49 mmol) was dispersed in extra dry THF (40 mL). The reaction vessel was then immersed in an ice bath and 4‐vinylbenzyl chloride (6.3 g, 37 mmol) was added dropwise with stirring. The mixture was heated to room temperature and then stirred for 24 h. The mixture was filtered to remove sodium chloride and 4‐vinylbenzyl methyl sulfide was recovered as a yellow oil after concentration under vacuum (90% yield). The obtained 4‐vinylbenzyl methyl sulfide was characterized by ^1^H NMR spectroscopy in CDCl_3_.

### Synthesis of the Zwitterionic Monomer

According to previous literature,^[^ ^21^ ^]^ zwitterionic monomer was prepared by ring‐opening of 1,3‐propyl sulfonic acid and 4‐vinylbenzyl methyl sulfide. 4‐vinylbenzyl methyl sulfide (4.5 g, 27 mmol) and BHT (0.25 g, 1.1 mmol) were dissolved in anhydrous acetonitrile (30 mL) and then 1,3‐propyl sulfonic acid (16.6 g, 136 mmol) was added. The mixture was stirred at room temperature until a homogeneous solution was formed, and then immersed in an oil bath (50 °C) and continued stirring for 67 h. The reaction was cooled down to room temperature and filtered to obtain a white solid, which was washed several times with THF and ether, and recovered by centrifugation. The final zwitterionic monomer (62% yield) was obtained by vacuum‐drying. The obtained zwitterionic monomer was characterized by ^1^H NMR spectroscopy in D_2_O.

### Preparation of Zwitterionic Polymer (PST) by Reversible Addition‐Fragmentation Chain‐Transfer (RAFT) Polymerization

Mercaptothiazoline‐activated trithiol‐RAFT agent (10 mg, 26 µmol), AIBN (1.0 mg, 6 µmol), zwitterionic monomer (300 mg, 1.05 mmol), and TFE (5 mL) were added to a 10 mL of round‐bottom flask. The flask was then sealed and the solution was degassed via four freeze pump‐thaw cycles. The polymerization was carried out at 70 °C for 10 h. The final mixture was dialyzed extensively against 0.5 M NaNO_3_ water solution using a dialysis tube with MWCO of 10 kDa, then against water to remove the salt. The obtained PST was characterized by ^1^H‐NMR spectroscopy in D_2_O.

### Synthesis of RITC‐Labeled HA, RITC‐Labeled GOx and RITC‐Labeled HRP

RITC‐labeled HA, GOx, or HRP biomolecule was synthesized by adding 2 µL of RITC DMSO solution (0.5 mg mL^−1^) dropwise into a stirred biomolecule aqueous solution (10 mg in 5 mL of PBS buffer pH 8.5). The mixed solution was stirred for 12 h. After that, the solution was dialyzed (dialysis tubing 10 kDa MWCO) extensively against Milli‐Q water and the product was obtained by freeze‐drying.

### Preparation of Coacervate Droplets and Membranized Coacervate

Preparation of stock solutions: (i) PST solution: 20 mg of PST was dissolved into 1 mL PBS buffer (10 mm) at different pH. (ii) Fe^3+^ ions solution: 27 mg mL^−1^ in H_2_O. (iii) Fe^2+^ ions solution: 19.8 mg mL^−1^ in H_2_O.

For simple self‐coacervation of PST studies, PST stock solution (20 mg mL^−1^) was prepared in PBS buffer (10 mm) and stored at 4 °C for 20 min. Phase separation occurred, with one phase being PST‐rich, considered the coacervate phase, and the other as the supernatant, a characteristic feature of coacervation. The dense phase was used for monitoring the formation of droplets by microscopic analysis.

For membranization of coacervate droplets studies, mixing the solutions of PST solution (20 mg mL^−1^) and Fe^3+^ ions solution (27 mg mL^−1^) or Fe^2+^ ions solution (19.8 mg mL^−1^) into an Eppendorf tube under different mass concentration ratios and pH.

### Transmittance or Turbidity Measurements

The UCST‐type phase transition of PST was measured upon cooling of a preheated solution. Specifically, transmittance at 500 nm of PST solutions (20 mg mL^−1^ in PBS, 10 mm, pH 5.0) at temperatures from 80 to 25 °C was measured on a PerkinElmer UV–Vis spectrophotometer (Lambda 750S, USA) equipped with a Peltier temperature controller. At each temperature, sufficient time (≈5 min) was allowed for equilibration.

PST (20 mg mL^−1^, PBS, 10 mm) and Fe^3+^ ions (27 mg mL^−1^, H_2_O) or Fe^2+^ ions (19.8 mg mL^−1^, H_2_O) solutions were mixed at different stoichiometries and acid‐base range (pH), in order to evaluate the coacervation behaviour of these components. Structural stability of coacervate droplets or membranized coacervate assemblages were also investigated, formed at different stoichiometries, toward different salt concentrations [NaCl], pH, and [urea] respectively. Measurements were performed on a PerkinElmer UV–Vis spectrophotometer. Turbidity was calculated as 100‐%T = 100‐(100^*^10^−Abs500^).

### Sequestration Properties

The sequestration property of the coacervate droplets were assessed by the difference at inner and outer of droplets from confocal laser scanning microscope images, respectively. 0.5 µL of molecules (RhB, RITC‐labelled HA, GOx and HRP) were added into 20 µL accomplished coacervate droplets. The fluorescence intensity of inner and outer of coacervate droplets were significantly distinguishing. The partition coefficient (*K*) was determined as *K = C_coa_/C_s_*, where *C_coa_* and *C_s_* were concentrations of the molecules in the coacervate droplets and solution phases, respectively. The fluorescence intensity was proportional to the concentration of molecular. Hence, *K* can be roughly determined as *K = I_coa_/I_s_*. The sequestration property of the membranized coacervate assemblages was carried out use the same method.

### Fluorescence Recovery After Photobleaching (FRAP)

FRAP experiments were performed on a laser scanning confocal microscope (Leica, TCS SP8) by bleaching a region of interest (ROI) of the microdroplets with a 488 nm laser with 100% energy intensity. After bleaching, a series of images were collected to analyze the recovery. Raw fluorescence data was obtained from Image J and normalized in Origin software.

### Complexation Rate of Fe^3+^ Ions with Coacervate Droplets

The complexation rate of Fe^3+^ ions with coacervate droplets was determined according to a 1,10‐phenanthroline spectrophotometric method.^[^ ^20^ ^]^ The detailed experimental procedure was as following: First, a standard solution of FeCl_3_ with different concentrations was prepared, and then 10 µL of FeCl_3_ solution was added to 100 µL hydroxylamine hydrochloride solution (10 wt.%, freshly prepared) and left to stand for 10 min. Subsequently, 100 µL 1,10‐phenanthroline (0.12 wt.%, freshly prepared) solution and 290 µL acetic acid‐sodium acetate buffer solution (pH 4.3, freshly prepared) was added, and the reaction was kept away from light for 15 min at room temperature. The absorbance at 511 nm was recorded and the standard curve of FeCl_3_ was plotted. After Fe^3+^ ions were complexed with PST, the supernatant was taken by centrifugation (6000 rpm, 10 min), and the Fe^3+^ ions content in the supernatant was detected according to the above steps. The complexation rate of Fe^3+^ ions with PST was calculated according to the standard curve.

### Determination of Hydroxyl Radicals (·OH) in Redox Reactions

The H_2_O_2_ can produce a large number of hydroxyl radicals (·OH) under the catalysis of trace Fe^2+^ ions. ·OH has strong oxidation properties and can quickly oxidize colorless TMB to blue Ox‐TMB.^[^ ^22^ ^]^ Briefly, RITC‐labeled GOx ‐containing (1 mg mL^−1^) coacervate droplets and Fe^2+^ ions (19.8 mg mL^−1^) were mixed at a volume ratio of 100:4. Subsequently, 1.0 µL of TMB (10 mg mL^−1^ in DMSO) was added into 100 µL of mixed coacervate droplets solution, then 50 µL of sample was added into the glass slide. The redox chemistry was initiated with the pipetting of 1 µL glucose (3 mg mL^−1^) into the sample cell and the fluorescence change was monitored by the CLSM every 10 s.

### HRP‐Mediated Peroxidation within Membranized Coacervate Assemblages

Typically, 5 µL of hydrogen peroxide (5 mm) was added to 100 µL of membranized coacervate assemblages encapsulating HRP (1 µL, 0.1 mg mL^−1^) and Amplex red (AR, 1 µL, 0.1 mg mL^−1^). The emission intensity of the product resorufin was monitored by CLSM over 800 s.

### Molecular Dynamics Simulations

A PST chain containing five repeating units using the GassView6.0 software was first conducted. To investigate the interactions between PST and PST, as well as PST and Fe^3+^ ions, two systems containing two PST, two PST, and two Fe^3+^ ions were constructed, respectively. These systems were solvated in water boxes (with the size of 4 nm × 4 nm × 4 nm) and neutralized with sodium ions, and then followed by a steepest descent minimization and a 100 ns NPT equilibration (300 K and 1 atm). After the equilibration of the simulation systems, a 100 ns production run in the MD (300 K and 1 atm) ensemble was performed for each system. Furthermore, the two systems were relaxed in an aqueous solution for 100 us to reach equilibrium (the fluctuation of internal energy in the system was less than 1 KJ mol^−1^), and the spherical structure obtained in the solution was defined as clusters. PST‐PST cluster contains two PST molecules and PST‐Fe^3+^ cluster contains two PST molecules and two Fe^3+^ ions. After the equilibration of the simulation systems, a 100 ns production run in the MD (300 K and 1 atm) ensemble was performed for each system. To understand the structural basis of the electrostatic surface potentials, the radial distribution functions, g(r), of Fe^3+^ ions and PST with respect to the center of the two types of clusters were calculated.

The AMBER99SB‐ILDN forcefield was used for PST, Fe^3+^ ions, and sodium ions. The SPCE model was chosen for water molecules. The periodic boundary conditions (PBC) were applied in all 3D. The long‐range electrostatic interactions were computed with the particle mesh Ewald (PME) method. The short‐range electrostatic interactions and the vdW interactions were truncated with a cutoff distance of 1.0 nm. The LINCS algorithm was adopted to constrain the bond vibrations involving hydrogen atoms, allowing for a time step of 2 fs. The system temperature (T = 300 K) was controlled using the velocity‐rescaled Berendsen thermostat. The pressure was set to 1 atm using an isotropic Parrinello‐Rahman pressostat. All the molecular dynamics simulations were conducted with the GROMACS package (version 2018.8) and VMD software (v1.9.3).

## Conflict of Interest

The authors declare no conflict of interest.

## Supporting information



Supporting Information

Supplemental Movie 1

Supplemental Movie 2

Supplemental Movie 3

Supplemental Movie 4

## Data Availability

The data that support the findings of this study are available from the corresponding author upon reasonable request.
