# Supplementary material for: Biomineralization‐Inspired Membranization Toward Structural Enhancement of Coacervate Community
Source: Adv Sci (Weinh). 2025 Mar 16;12(18):2417832. doi: 10.1002/advs.202417832 (PMC12079539; doi:10.1002/advs.202417832)
Supplement: Supplementary file 1 — Supporting Information [file ADVS-12-2417832-s002.docx]

**Biomineralization-Inspired Membranization towards Structural Enhancement of Coacervate Community**

Chunyu Zhao,^[a]^ Xiaoliang Wang,^[b]^ Lianning Li,^[c]^ Hu Huang,^[c]^ Bingzhao Wu,^[c]^ Lei Zhang,*^[^^c]^ and Xin Huang*^[b]^

Supplementary Figures


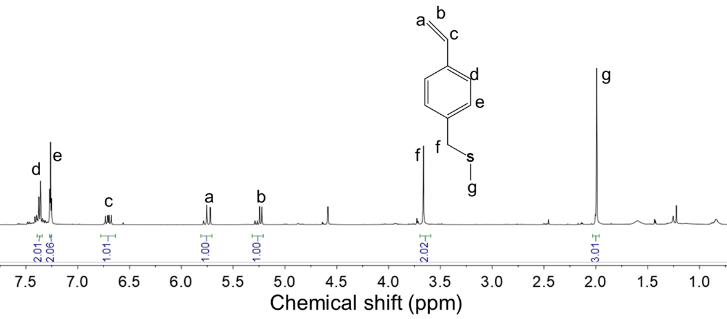


**Figure S1.** ^1^H-NMR spectrum of the 4-vinylbenzyl methyl sulfide in CDCl_3_.


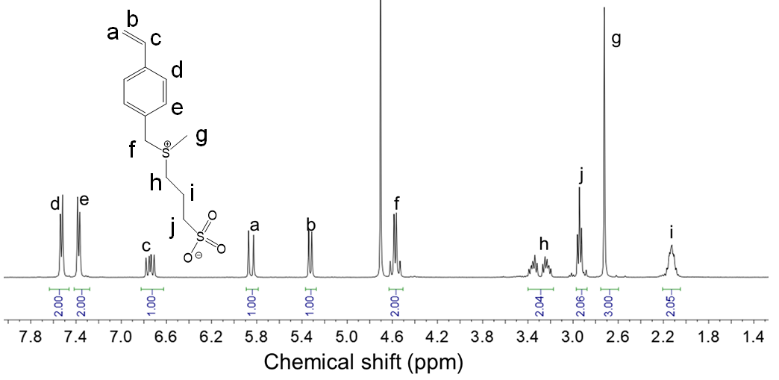


**Figure S2.** ^1^H-NMR spectrum of the zwitterionic monomer in D_2_O.


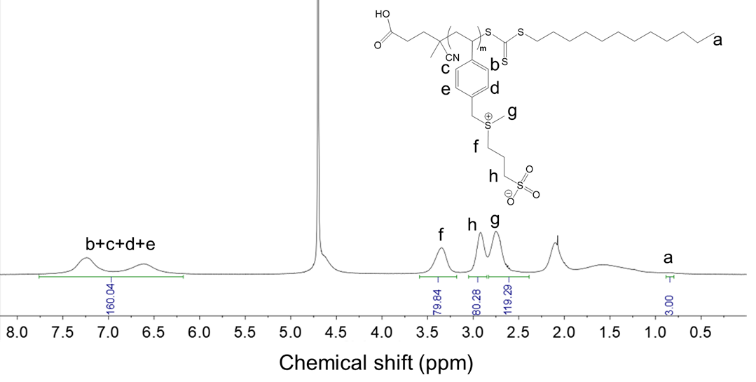


**Figure S3.** ^1^H-NMR spectrum of the PST in D_2_O.


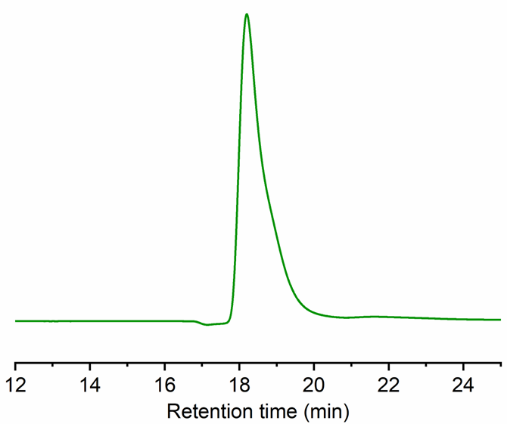


**Figure S4.** GPC profile of PST using H_2_O as solvent.


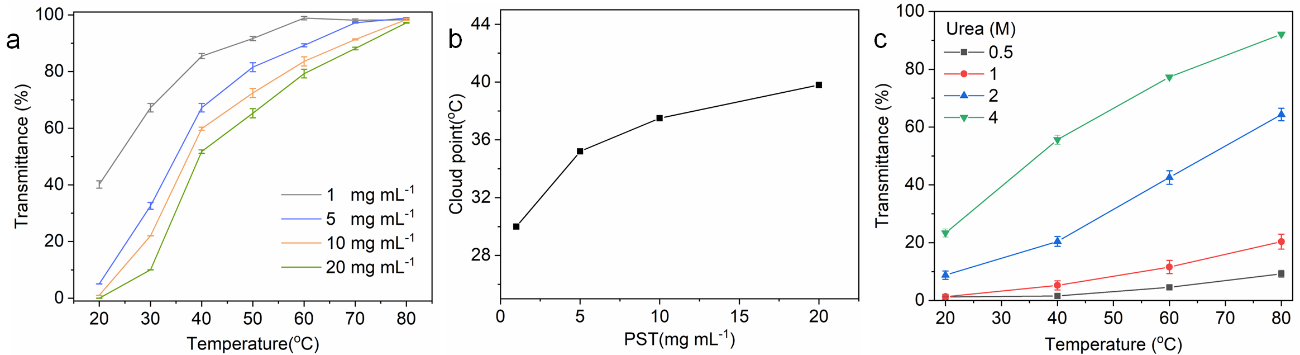


**Figure S5.** UCST behavior of PST in phosphate buffer solution (10 mM, pH 5.0). (a) Evolution of solution transmittance as a function of temperature upon cooling of PST solutions. (b) Variation of cloud point temperature of PST at different concentrations. (c) Influence of urea on UCST characteristics of PST (concentration = 20 mg mL^-1^).


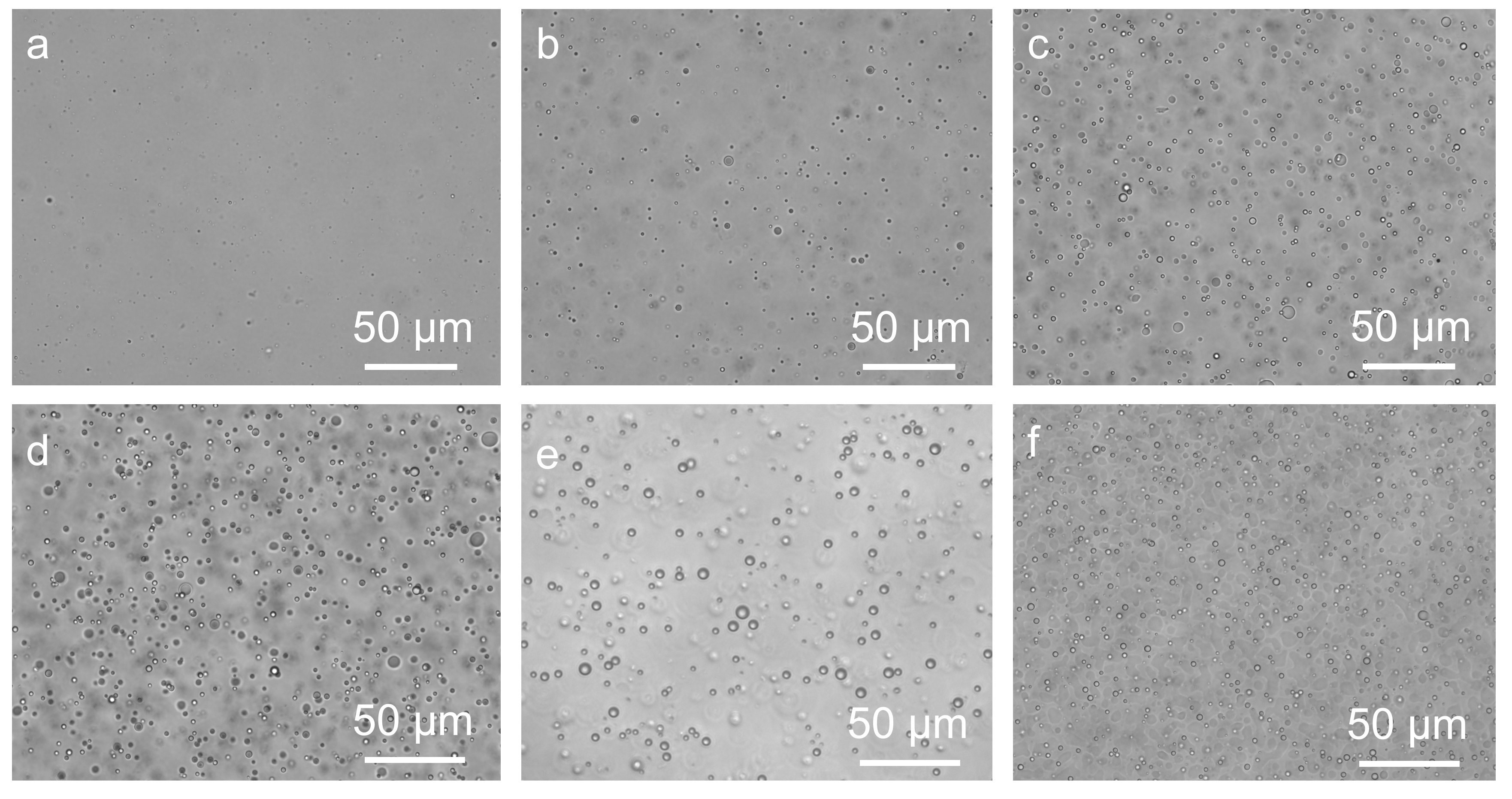


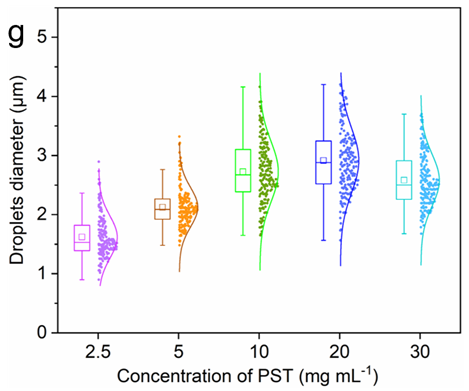


**Figure S6.** Optical microscopy images of the coacervate droplets prepared at different concentrations of PST solution, (a) 2 mg mL^-1^, (b) 2.5 mg mL^-1^, (c) 5 mg mL^-1^, (d) 10 mg mL^-1^, (e) 20 mg mL^-1^, (f) 30 mg mL^-1^ and (g) the corresponding diameter distribution of coacervate droplets. Approximately 300 droplets were measured with Image J.


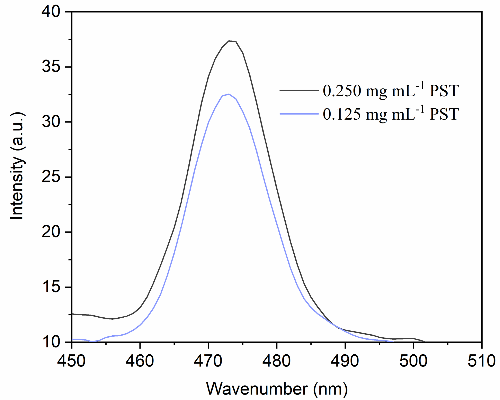


**Figure S7.** The fluorescence spectrum of the PST solution under different concentrations, and the excitation wavelength was 405 nm.


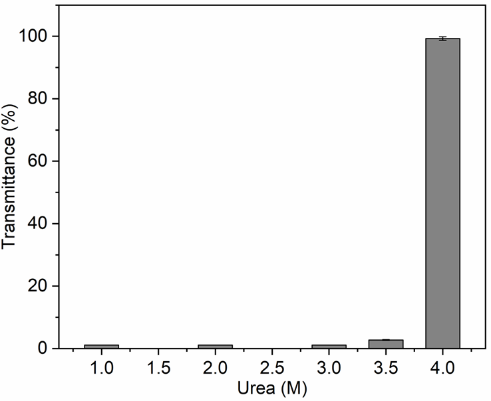


**Figure S8.** Transmittance measurements of the coacervate droplets (PST, 20 mg mL^-1^) at different urea concentrations. Error bars indicate the standard deviation of three replicating measurements.


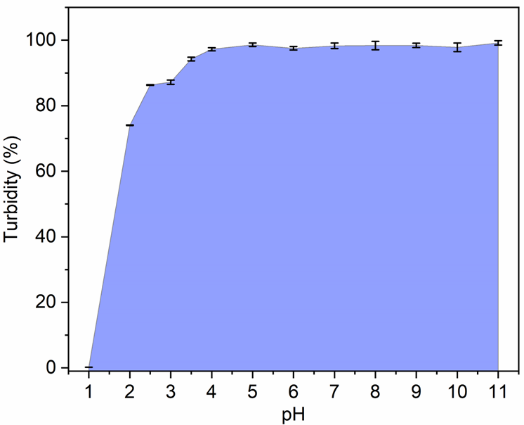


**Figure S9.** Turbidity measurements of the coacervate droplets (PST, 20 mg mL^-1^) at different pH. Error bars indicate the standard deviation of three replicating measurements.


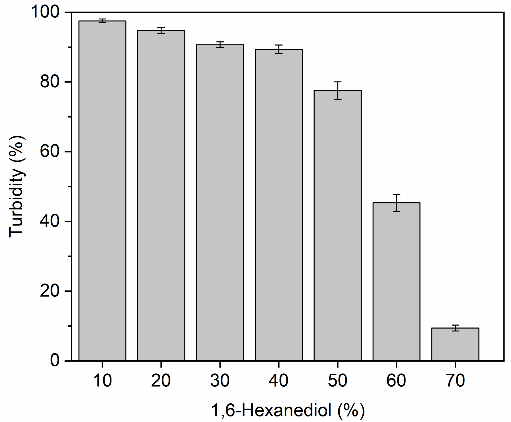


**Figure S10.** Turbidity measurements of the coacervate droplets (PST, 20 mg mL^-1^) at different 1,6-hexanediol concentrations. Error bars indicate the standard deviation of three replicating measurements.


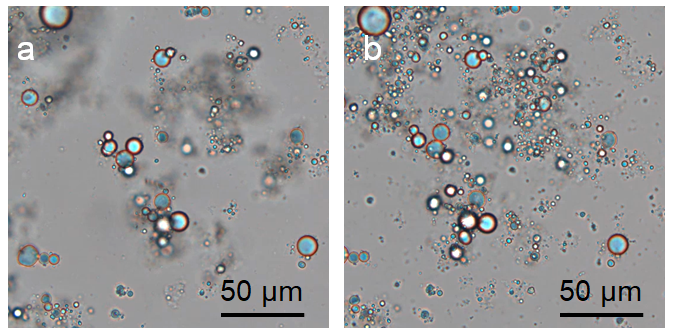


**Figure S11.** Optical microscopy images showed the stability of coacervate assemblages in situ in 60 min, (a) t_0_+0 min, (b) t_0_+60 min.


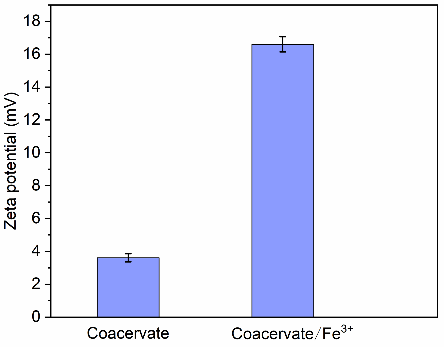


**Figure S12.** Zeta potentials of the coacervate droplets (PBS 10 mM, pH 5.0), Fe³⁺ ions solution (100 mM, pH 5.0), and the PST (1.69 mM, PBS 10 mM, pH 5.0) with Fe^3+^ ions (PST: Fe^3+^, molar ratio, 1:1.8) were determined at room temperature. All relevant experiments were performed independently at least three times with similar results.


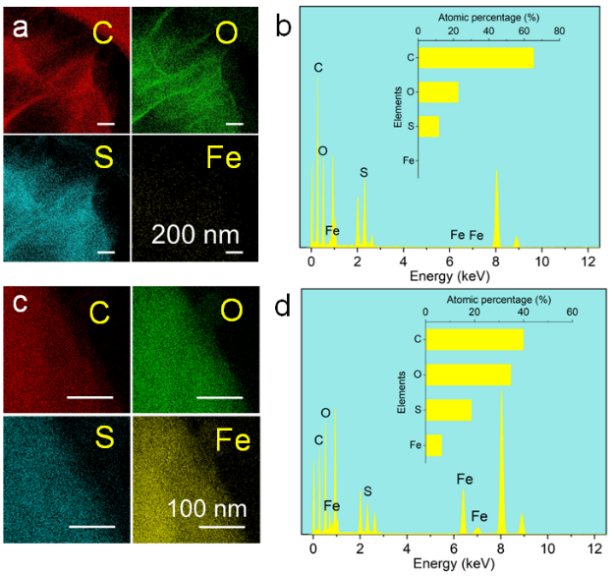


**Figure S13.** EDX elemental mappings and the atomic percentage of C, O, S and Fe on the surface of (a, b) the coacervate droplets and (c, d) membranized coacervate assemblages.


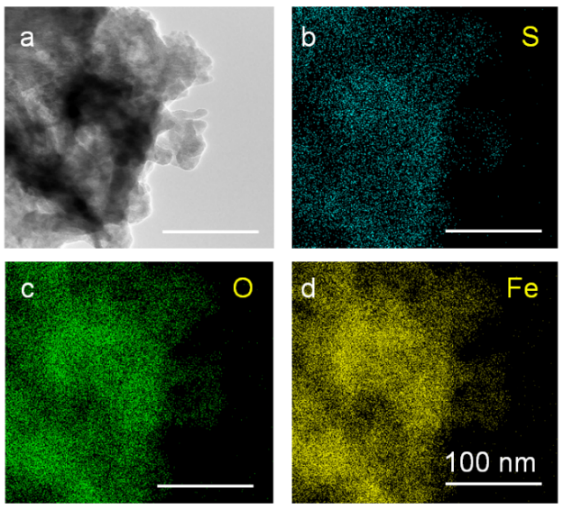


**Figure S14.** The HR-TEM images of the partial enlarged detail of (a) the blue area and the corresponding EDX elemental mappings of (b) S, (c) O and (d) Fe.


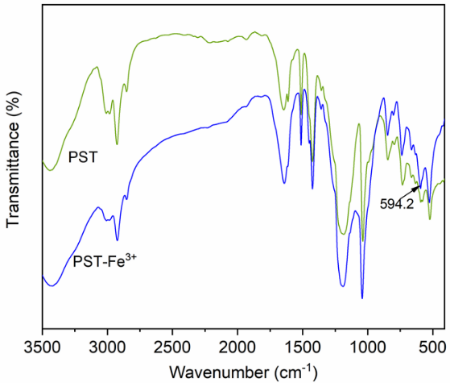


**Figure S15.** FTIR spectra of PST and dried coacervate assemblages (PST-Fe^3+^).


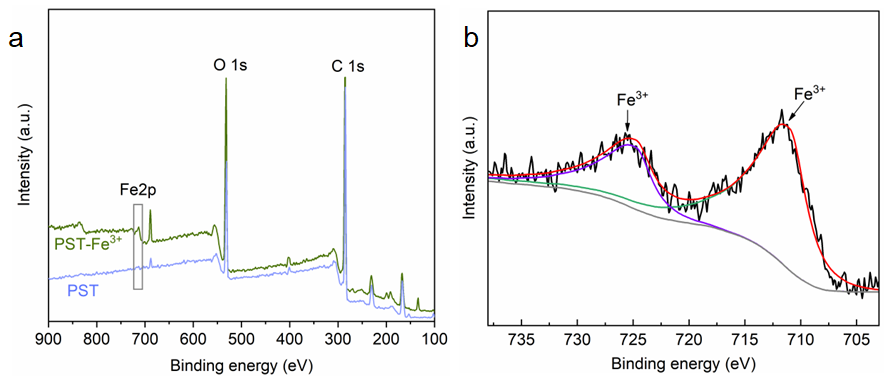


**Figure S16.** XPS spectra of PST and dried coacervate assemblages (PST-Fe^3+^), (a) full spectrum, (b) Fe 2p in dried coacervate assemblages.


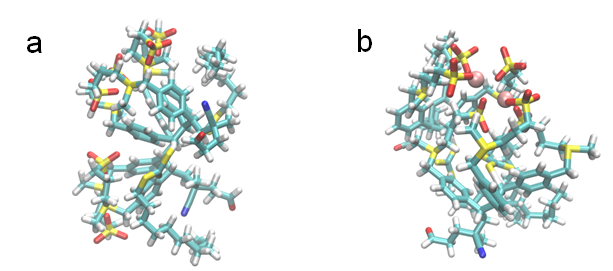


**Figure S17.** Representative snapshots of (a) PST binding to PST and (b) Fe^3+^ ion binding to PST. The carbon atom, oxygen atoms, nitrogen atoms, hydrogen atom, sulphur atom and iron atom are specifically shown in cyan, red, blue, white, yellow and red-orange, respectively.


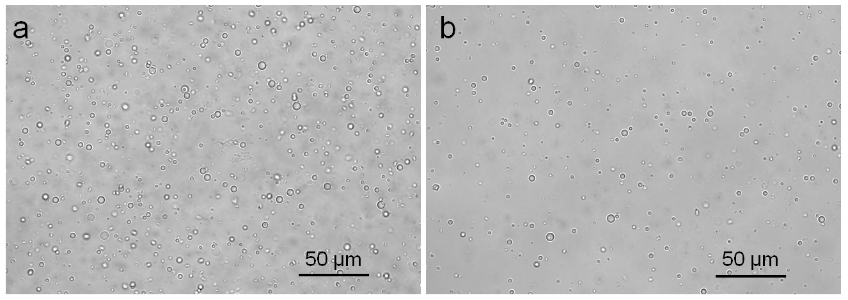


**Figure S18.** Optical microscopy images of the coacervate droplets with Fe^3+^ at pH 2.0, (PST: Fe^3+^, molar ratios, (a) 1:3, (b) 1:6.


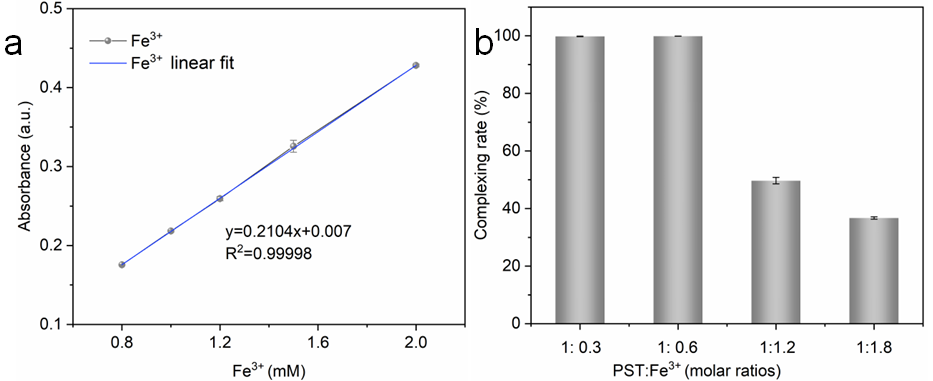


**Figure S19.** The complexation rate of Fe^3+^ in coacervate assemblages at pH 2.0. (a) Calibration curve for Fe^3+^ determined by plotting the UV-vis absorbance at 511 nm against concentration. (b) The complexation rate of Fe^3+^ with PST under different mass concentration ratios.


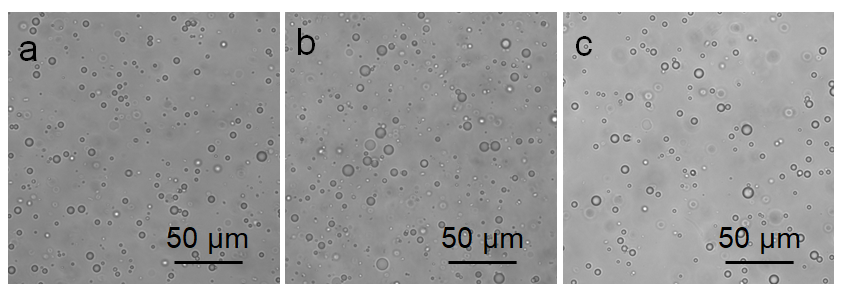


**Figure S20.** Optical microscopy images of the PST: Fe^2+^ under different molar ratios at pH 5.0, (a) 1:0.6, (b) 1:3, (c) 1:6.


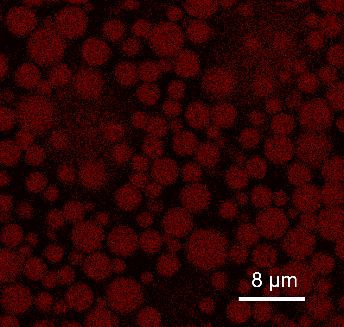


**Figure S21.** The CLSM images of the RITC-labeled GOx in the coacervate assemblages at t_0_+30 min.


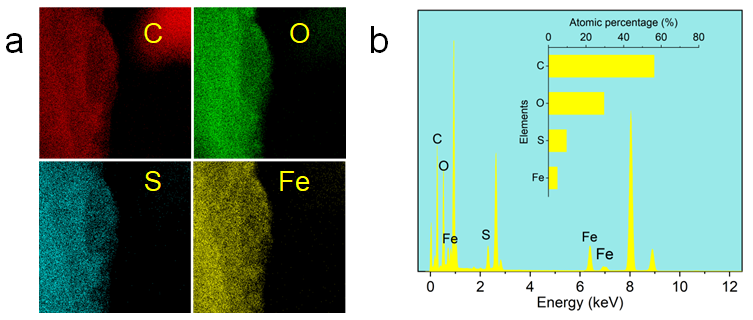


**Figure S22.** (a) EDX elemental mappings of C, O, S and Fe on the surface of the coacervate assemblages and (b) the atomic percentage at t_0_+15 min.


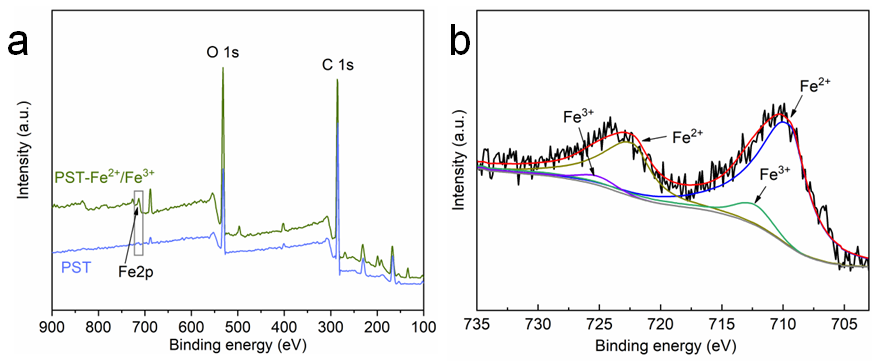


**Figure S23.** XPS spectra of PST and dried coacervate assemblages (PST-Fe^2+^) at t_0_+15 min, (a) full spectrum, (b) Fe 2p in dried coacervate assemblages.


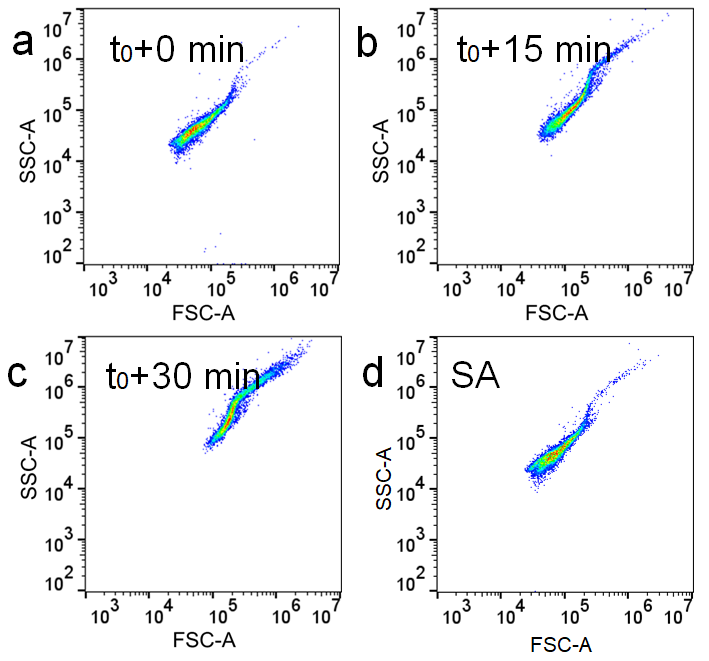


**Figure S24.** Fluorescence-activated cell-sorting time-series of FSC-A versus SSC-A dot plots for the PST-Fe^2+^ coacervate droplets by redox chemical, (a) t_0_+0 min, (b) t_0_+15 min, (c) t_0_+ 30 min, and (d) 2D pseudocolor plots for the re-dispersed individual droplet by added SA.


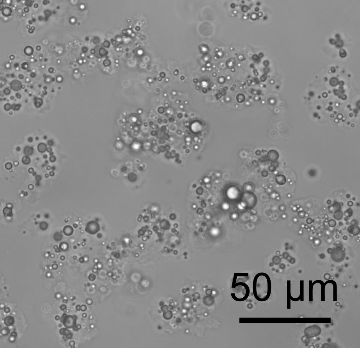


**Figure S25.** Optical microscopy image of the coacervate droplets (PST, 20 mg mL^-1^, pH 8.0) with FeCl_2_ (100 mM, H_2_O), (PST: Fe^2+^, 1:2.4, molar ratio).


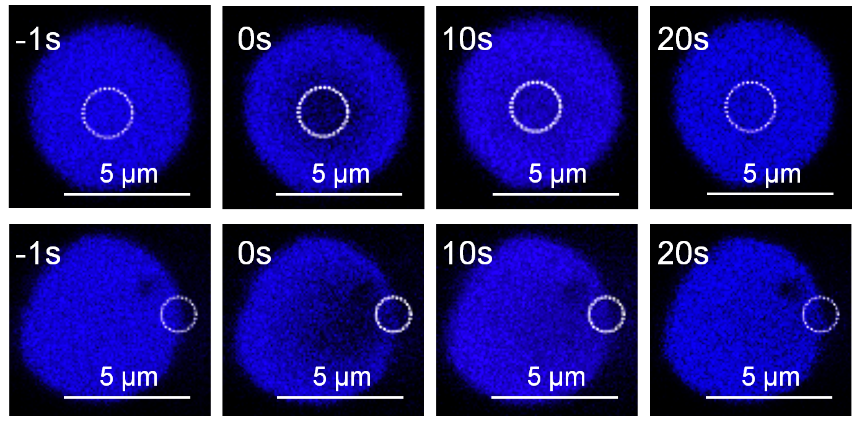


**Figure S26.** Time series of CLSM images after photobleaching the area (the core region or the edge region) in coacervate droplets (intrinsic fluorescence)


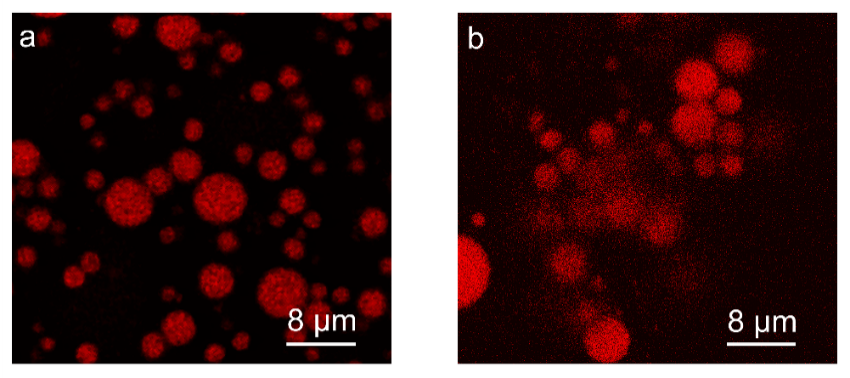


**Figure S27.** The CLSM images of produced resorufin in (a) coacervate droplets and (b) coacervate assemblages, respectively.


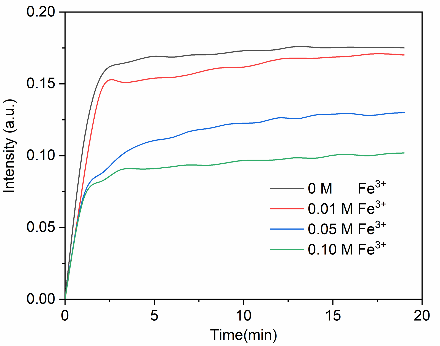


**Figure S28**. Fluorescence spectra of the RS by H_2_O_2_-mediated oxidation catalyzed by HRP without coacervates under different concentration of FeCl₃ (excitation wavelength 552 nm, emission wavelength 610 nm).


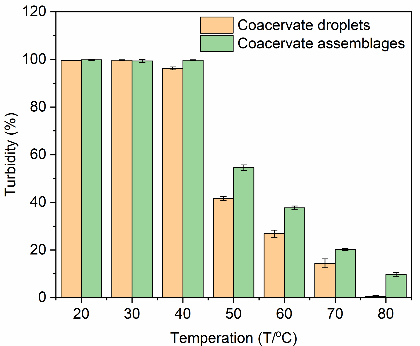


**Figure S29.** Turbidity measurements of the coacervate droplets and membranized coacervate assemblages at different temperature (equilibrate at each temperature for five minute). Error bars indicate the standard deviation of three replicating measurements.


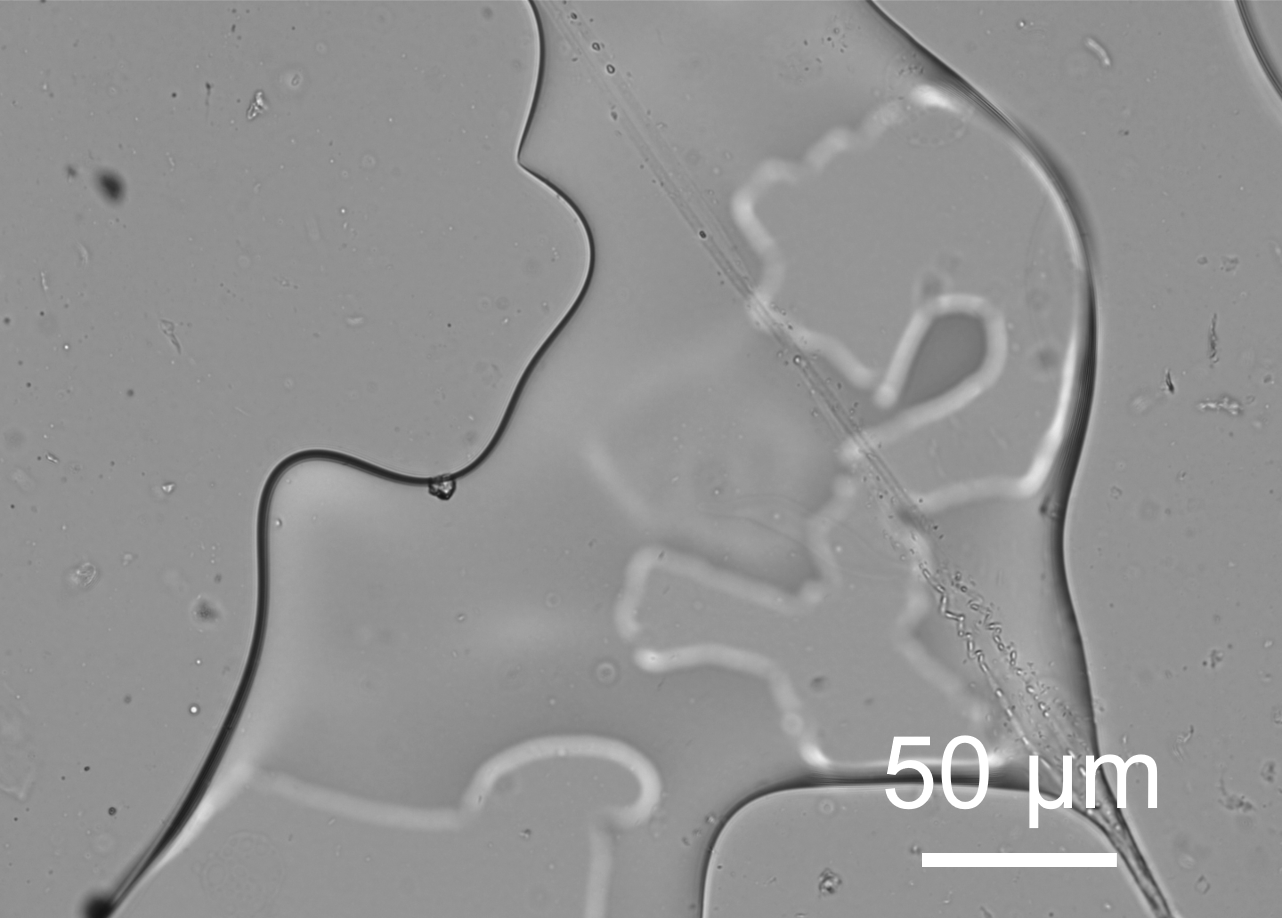


**Figure S30.** Optical microscopy image of the coacervate droplets had already coalesced at lower centrifugation conditions (3000 rpm, 5 min).


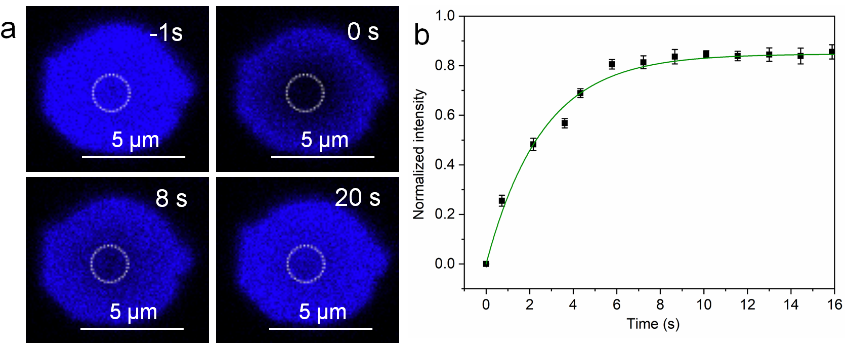


**Figure S31**. (a) Time series of CLSM images after photobleaching a confined area in membranized droplets (250 mM NaCl, intrinsic fluorescence) and (b) corresponding fluorescence recovery curves in bleached areas. Error bars represent standard deviations of three measurements.


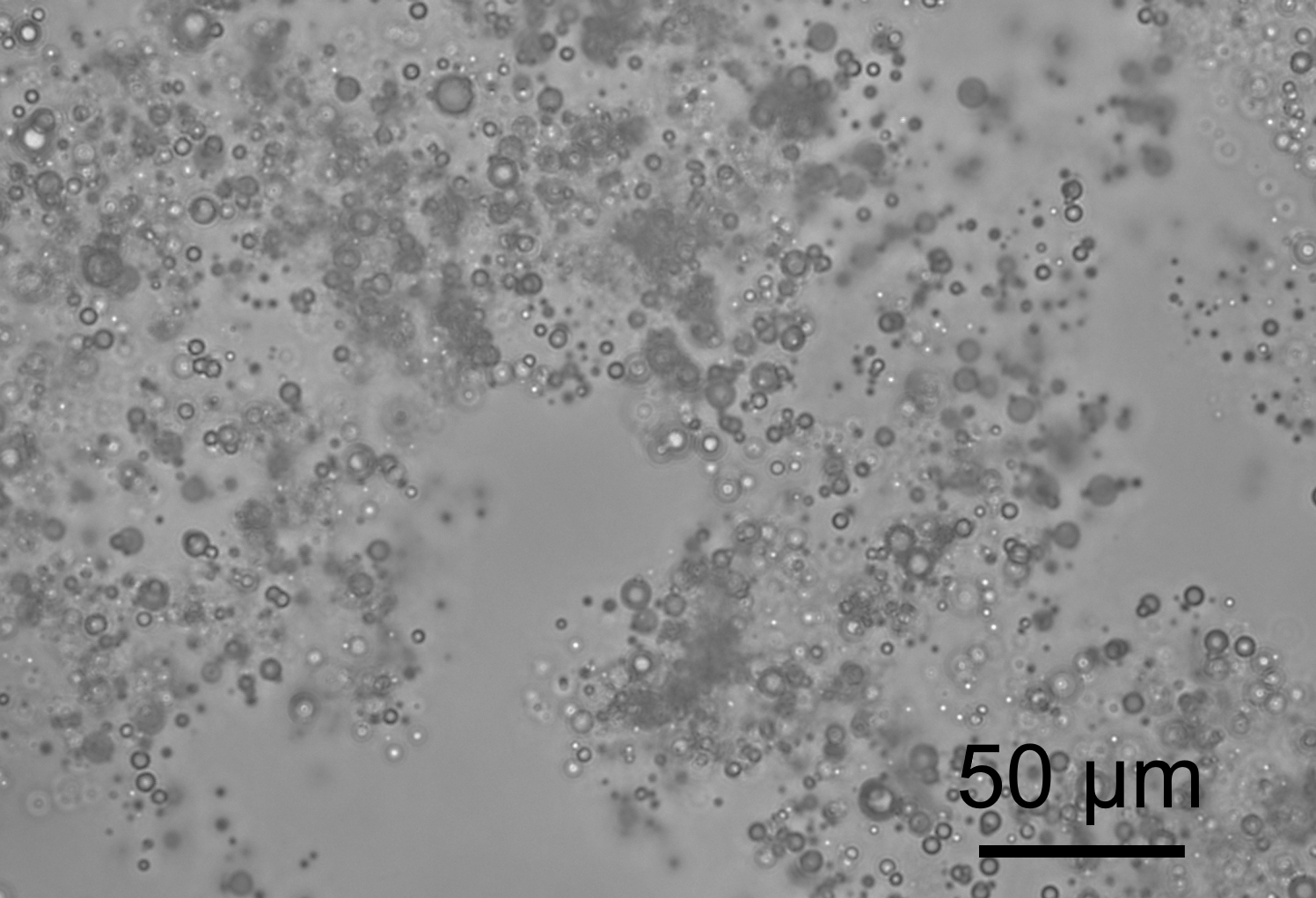


**Figure S32.** Optical microscopy image of the coacervate assemblages after 30 days.

**Supplementary Movie S1**

Adding the PBS buffer into the coacervate assemblages solutions. The movie speed 10x.

**Supplementary Movie S2**

The coacervate assemblages moved as a single unit under gentle stirring. The movie speed 1x.

**Supplementary Movie S3**

The aggregation processes of the GOx-loaded coacervate droplets (PST-Fe^2+^ ions) by adding the glucose in situ. The movie speed 10x.

**Supplementary Movie S4**

The re-dispersion processes of the coacervate assemblages by adding the SA in situ. The movie speed 10x.
